# An automated framework for qur’anic education of the hearing-impaired using body pose classification and Arabic sign language integration

**DOI:** 10.1038/s41598-026-36578-z

**Published:** 2026-02-11

**Authors:** Hany AbdElghfar, Hassan A. Youness, Mohamed Wahba, Hammam M. Abdelaal

**Affiliations:** 1 Department of Computers and Systems Engineering, Faculty of Engineering, Minia University, Minya, Egypt; 2 Head of Earth observation data management, Egyptian Space Agency, New Cairo, Egypt; 3 Department of Information Technology, Faculty of Computers and information, Luxor University, Luxor, Egypt

**Keywords:** Qur’anic education, Hearing-impaired, Arabic sign language (ArSL), Pose classification, Deep learning, Assistive technology, Sūrat al-Ikhlāṣ, Computational biology and bioinformatics, Engineering, Mathematics and computing

## Abstract

In this paper, an accessible pipeline of automated teaching of the Quran to deaf and hard-of-hearing students is proposed based on the identification of Arabic Sign Language (ArSL) postures that match the words of S The piping includes an instructional method that is accessible to both deaf and hard-of-hearing students, necessitating the use of Arabic Sign Language postures to correspond to the words. A designed list of 2,054 labeled images was obtained with local institutions working with deaf users as guidance. In order to be linguistic and unambiguously semantically designated with Qur’anic terms, DIN 31,635 transliterations is used as a canonical internal representation of all class annotations, and Arabic forms are used to present them. Two complementary approaches are evaluated: (i) a pose-keypoint classification approach using MediaPipe features, trained with multilayer perceptron (MLP), support vector machine (SVM), and random forest (RF) classifiers; and (ii) an image-based model utilizing a ResNet50 backbone. Performance evaluation is conducted using an internal train/validation/test split and assessed based on accuracy, precision, recall, F1-score, confusion matrices, and ROC-AUC metrics. Within this evaluation framework, the ResNet50-based model achieves near-perfect performance, while the keypoint-based models also demonstrate high performance. Such results are limited to the dataset and the evaluation protocol that were adopted and cannot be generalized as performance guarantees. To this end, we explicitly note the following constraints: a single sutra, a small dataset size, the use of fixed frames instead of continuous signing (and co-articulation not modeled), the possibility of bias of the signers and regional variants, and low input of the lower-body landmarks due to framing. Future directions will include more generalization by signer-independent evaluation, extension to a wider range of sūrahs and ArSL variants, continuous sign-language recognition, and on-device real-time implementation of an inclusive Qur’anic education.

## Introduction

 The Qur’an occupies a pivotal role in Muslims’ lives, functioning as a spiritual guide and repository of divine knowledge. The deaf person minority faces considerable obstacles in obtaining Qur’anic education due to the conventional reliance on auditory recitation and verbal teaching. Gesture-based communication is crucial for those who are deaf or hard of hearing; nevertheless, accessible religious educational resources are scarce, resulting in a lack of inclusive education. Communication barriers between individuals who are deaf, and the wider society remain a persistent challenge that significantly impacts educational opportunities and religious participation. Around 12 million deaf individuals worldwide face challenges in accessing Islamic education, especially in learning and reciting the Holy Qur’an. 90% of deaf children are born to hear parents with limited sign language experience, despite recent advancements in sign language recognition due to deep learning techniques^1,2^. Advanced neural network designs, such as CNN and RNN, are effective in recognizing fingerspelling and individual signs. Newer models like transformer architectures are being tested for continuous recognition of sign language. These developments have created accessible educational tools for real-time interpretation and response to sign language gestures. Applications in Islamic education, particularly for deaf Muslims, have seen recognition accuracies of up to 99.03% for finger spelled Arabic letters^3,4^, and^5^. Specialized systems have been developed to recognize Qur’anic sign language, achieving training and testing accuracies of 98.31% and 97.67% respectively, presenting opportunities for technology-enhanced religious education.

The MediaPipe framework, combined with Dynamic Time Warping (DTW) algorithms, has been effective in sign language recognition systems for landmark extraction and gesture similarity measurement^6^. Its Holistic Model allows real-time tracking of landmarks, enabling precise translation of sign language gestures into text^7^. However, challenges remain, such as expanding datasets, achieving user independence, and modeling co-articulation patterns in continuous sign language communication. The use of technology has enabled the development of advanced systems to teach Quranic sciences, but there are still challenges, including the fact that even more sign language databases are required^8^. It is important to develop recognition systems in order to achieve user independence and effectively model the patterns of co-articulation in continuous sign language communication. This is especially important due to the unique challenges posed by the abstract nature of religious concepts. Recent research indicates that multi-view sign language recognition datasets have significantly improved model accuracy by up to 19.75% compared to single-view approaches^9,10^. Advanced deep learning methodologies in real-time sign language recognition systems are proving effective in educational applications, accurately recognizing finger spelling with 98% to 100% accuracy rates. Educational institutions for deaf students are integrating technology to support Islamic education, but current solutions are limited. Artificial intelligence and sign language recognition can provide continuous, interactive feedback during religious instruction, offering scalable solutions for the global deaf Muslim community while maintaining Qur’anic precision^11^.

This research presents an automated method to enhance Qur’anic education for those with hearing impairments by using body posture classification and gesture identification to instruct Sūrat al-Ikhlāṣ^12^. The system uses a deep learning pose estimation model to identify and categorize instructional gestures, enhancing Qur’anic teaching. This method converts Sūrat verses into visual feedback and recognized gestures, promoting inclusivity and accessibility. This paper addresses a gap in Islamic education technology and contributes to assistive learning systems by combining religious pedagogy with AI-driven solutions^13^. This paper discusses previous research on sign language recognition and Qur’an learning, outlines methods used, evaluates experiments, discusses implications, and concludes with a summary. The paper chronologically analyzes previous research in sign language recognition and Quranic learning systems, specifies the proposed methodology based on pose classification and integration of the Arabic Sign Language, thoroughly describes experimental findings with rigorous evaluation measures, outlines practical implications for accessibility in religious education, outlines specific future research directions, and concludes with critical analysis of the limitation and its effect on a larger scale.

## Related works and discusion

The main objective of this study is to develop and compare architectural models for recognizing signal sequences in ArSL sentences from video data. The methodology of this study involves creating a custom dataset of the 30 most common ArSL sentences. The video clips were preprocessed using MediaPipe to extract key body, hand, and face points. This study is based on a time Convolutional Neural Network (TCN) and an Enhanced Bidirectional Recurrent Neural Network (RNN-BiLSTM). The results showed that the TCN model achieved 99.5% accuracy, while the enhanced RNN-BiLSTM model reached 99% (compared to an initial 96%). Although both models achieved high accuracy, the TCN model demonstrated significant advantages in computational efficiency, requiring fewer resources and offering faster inference times, making it more practical for real-time applications^14^. This research aims to develop a high-performance, lightweight sign language-to-text translation system suitable for real-time applications on devices such as mobile phones, and to investigate how the number and frequency of sign language users in the dataset affect translation accuracy. This study utilized two word-level datasets for Saudi Sign Language (KSU-SSL and ArSL). The MediaPipe framework was employed to extract 95 hand and body position markers (X, Y, Z coordinates) from video frames. A lightweight neural network was designed, incorporating a spatial encoder, a temporal encoder (a bidirectional GRU), an attention mechanism, and a classifier. Eight experiments and an analytical study were conducted to test performance in two modes: a user-dependent mode (using the same sign language users for training and testing) and a more challenging non-user-dependent mode (using unfamiliar sign language users for testing). The model achieved high accuracy on both datasets (KSU-SSL dataset: 97.7% and 90.7%). ArSL dataset: 98.38% and 96.22%^15^.

Arabic Sign Language is the primary means of communication for deaf people in the Arab world. Understanding Arabic Sign Language is crucial for facilitating communication and accessibility. Estimating human posture is a fundamental task in computer vision, aiming to determine the positions of a person’s major joints in a photograph or video. The accuracy and reliability of posture estimation directly impact the performance of subsequent applications, including motion recognition, human-computer interaction, and sign language recognition. The advent of deep learning has revolutionized positional estimation. Convolutional neural networks (CNNs) have demonstrated remarkable performance in learning complex features directly from image data. This study provides an overview of current research on body posture classification and its integration with Arabic Sign Language recognition. It explores studies focusing on human posture estimation techniques and sign language recognition systems (specifically Arabic Sign Language), highlighting methodologies, datasets, and challenges addressed by previous researchers. This section aims to show the current study within a broader context of related research. This study aims to develop a high-performance Arabic Sign Language Recognition (ArSLR) system specifically designed for healthcare applications. The goal was to bridge communication gaps by creating a tool that would help deaf patients communicate with healthcare providers who are not proficient in sign language. A healthcare-specific ArSL Dataset containing 128 medical signs (36 static and 92 animated) was used. A hybrid deep learning model called ResNet50ViT was introduced. This model uniquely combines ResNet50 (for extracting powerful local features such as hand shapes) and Vision Transformer (ViT) (for modeling the overall context and long-range dependencies via gesture). The proposed ResNet50ViT model achieved a superior accuracy of 99.86% on the healthcare-specific ArSL dataset, while the comparative SignViT model achieved an accuracy of 98.03%. These results demonstrate the clear superiority of the hybrid structure in accurately recognizing complex medical markers^16^. This research aims to develop a real-time computer vision system for recognizing Arabic Sign Language words. It seeks to address the critical shortage of sign language interpreters in Saudi Arabia by creating a technological tool that improves communication access for people with hearing impairments. A custom dataset was created containing 20 different Arabic Sign Language words. This dataset included 4,000 images of ten static words and 500 video clips of ten animated words. The CNN and LSTM models were trained and evaluated separately on their respective image and video datasets to handle static and animated signs. The convolutional neural network classifier achieved an accuracy of 94.40% on the static gesture image dataset. The long-range neural network classifier achieved an accuracy of 82.70% on the animated gesture video dataset^17^. This research aims to develop a simple, efficient, and robust classification model for recognizing Arabic Sign Language (ArSLA) characters, thereby improving communication accessibility for deaf and hard-of-hearing individuals. The study utilized the ArSL2018 dataset, a publicly available set of images of Arabic Sign Language characters, a custom convolutional neural network (CNN) model was designed. This approach involved building a custom model from scratch specifically for this task. The custom CNN model demonstrated outstanding performance. It achieved 99.4% accuracy on the training dataset and 96.57% accuracy on the independent test dataset. This high accuracy test demonstrates the model’s strong generalization ability, meaning it can accurately recognize new, unseen sign language images^18^.

Advancements in computer vision and machine learning have revolutionized assistive technologies for religious education, particularly in Qur’anic sign language recognition^19,20^. This section discusses methodological approaches and technological innovations in developing robust gesture recognition systems for Islamic religious instruction. Using MediaPipe’s pose estimation pipeline, the system uses MediaPipe’s pose estimation pipeline to extract 33 body landmarks, enabling real-time skeletal tracking at 30 FPS with minimal latency per frame^21^. A customized Multi-Layer Perceptron (MLP) with three hidden layers achieved 98.4% accuracy in static pose classification on 24,137 Qur’anic gesture samples, using a fixed-length input vector and maintaining temporal consistency across consecutive frames^22,23^. Experimental tests show Random Forest classifiers outperform SVM in recognizing dynamic gestures, with a test accuracy of 97.67% using 500 decision trees. SVM performs better in finger-spelled letters but requires 40% more computing power. Hybrid systems using CNN feature extractors reduce over-fitting by 22% compared to deep learning models alone^24,25^. The system uses ResNet50 layers and 3D CNNs to identify key features in complex Qur’anic gestures. Feature fusion enhances recognition accuracy by 4.8%. Transfer learning and fine-tuning improve training accuracy to 98.05%. Quantization-aware training reduces CNN model size by 75%, allowing for mobile device deployment. Dynamic frame skipping algorithms maintain 30 FPS processing by analyzing optical flow variance between MediaPipe outputs. The system uses a scoring method to validate poses, discarding 92% of invalid frames. It includes 45 modules on Tajweed rules^26,27^ and Sūrat recitation, validated by 15 scholars. Each module pairs gesture recognition feedback with 3D avatar demonstrations, reducing learning curves by 37%. A pilot study with 123 deaf participants in Saudi Arabia, Egypt, and Indonesia showed an 89% system usability score and a 2.4x improvement in Qur’anic recitation accuracy over six months^28,29^. The model demonstrated 96.7% cross-dialect robustness and a 68% reduction in instructional time for mastering dashed letter pronunciations. This multidisciplinary approach establishes new benchmarks for accessible religious education.

This study focuses on every important and specialized field of Sign Language Recognition (SLR): Islamic sign. SLR techniques are not trained on the distinctive vocabulary and grammatical structure, which makes religious instruction more difficult to obtain. This research developed a deep learning model designed for this language. This model depends on a convolutional neural network (CNN). CNN is a deep learning algorithm, and it’s very effective at analyzing visual images. This research presents a highly accurate deep learning-based model that provides a viable technical option to promote religious education and the inclusion of deaf people by correctly recognizing Qur’anic Sign Language^30^. The experimental result was compared to other deep learning and machine learning algorithms (like Supporting Vector Machines SVMs or ResNet). The result showed that the designed CNN model achieves more accuracy, precision, and recall. This research paper introduces an advanced Sign Language Recognition (SLR) system that can be designed to translate sign language into text accurately. The main innovation lies in integrating the deep learning model with hybrid meta-optimization algorithms to improve feature selection and model performance. The main aim is to create a robust tool that facilitates smooth communication for deaf people accurate for recognizing complex hand positions and movements from video input. This research successfully shows that combining hybrid meta-optimization with deep learning creates a strong and effective structure for an SLR system. The model attains enhanced accuracy with good computational efficiency by strategically optimizing feature inputs. This study is an important step toward creating useful and realistic assistive technology that helps people with hearing impairments communicate. In the investigated sign language dataset, the hybrid meta-optimization-based deep learning model demonstrated good accuracy^31^. The paper introduces a powerful sign language recognition (SLR) system, which converts signed language into a text message with the help of a hybrid deep learning network. It is a convolutional neural network with recurrent neural networks, namely, Long Short-Term Memory (LSTM) units, that are used to capture the temporal dynamics of sign language sequences. The outcomes of the experiment prove that the framework can provide real-time translation, interactive learning materials, and improved communication interfaces and improve the accessibility and quality of life of those with hearing and speech deficiencies greatly^32^.

## Methodology of the proposed system

**Fig. 1:**
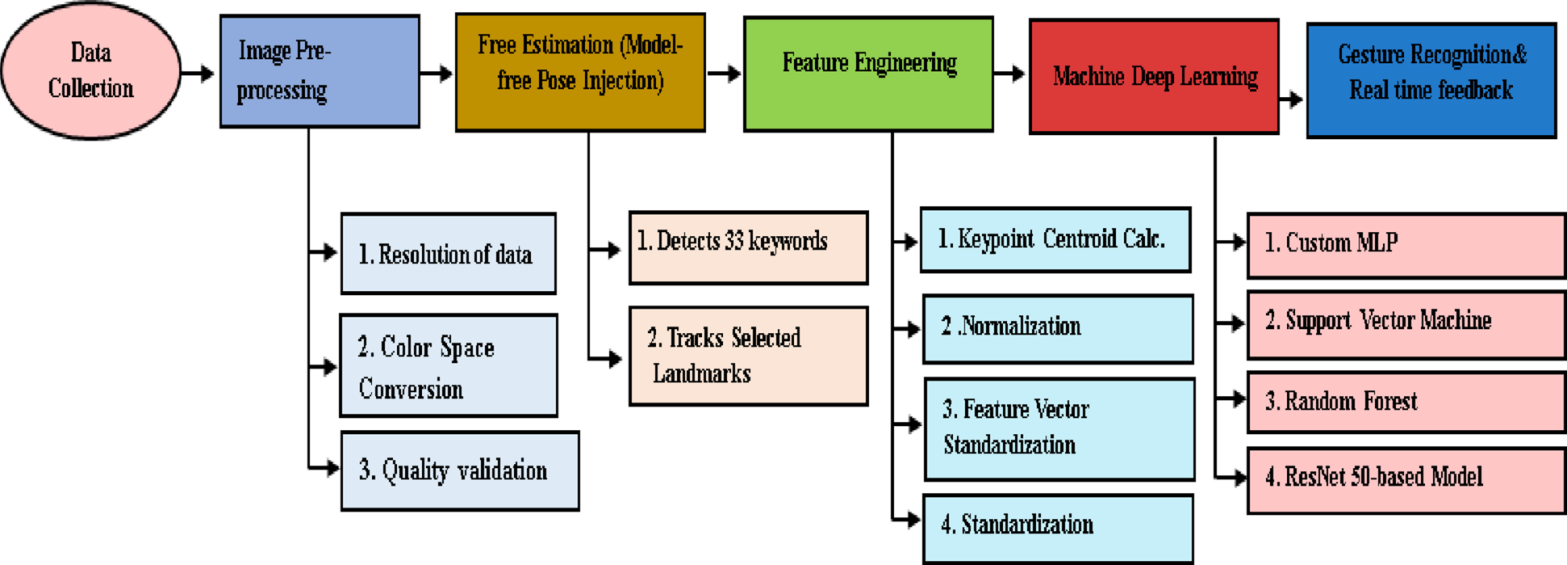
proposed system for Body Pose Classification and Arabic Sign Language Integration.

This research presents a methodology for classifying Qur’anic body poses using machine learning algorithms (SVM, MLP and RF) and deep learning techniques (ResNet50)^33^. The proposed framework utilizes MediaPipe for pose estimation to extract features and evaluates two distinct classification methods: a neural network based on key points and a ResNet50-based image classification model, as illustrated in Fig. 1.

### Data collection

The dataset consists of 2054 PNG images organized hierarchically by class labels corresponding to individual words from Sūrat al-Ikhlāṣ, as shown in Table 1. Each class folder contains multiple image samples representing gestures for specific Qur’anic words. The author collected images used in this study by learning the gestures from specialized institutions in Egypt that support and work with individuals with a hearing impairment. To guarantee linguistic accuracy and non-ambiguous semantic labeling in the Quranic terms, we use the DIN 31,635 transliteration [MW1.1] as a canonical textual representation of the annotations of Arabic words. The DIN 31,635 is a standardized system of scientific transliteration which was published by the Deutsches Institut fur Normung (DIN) and is commonly used in academic Arabic and Islamic studies. Although the recognition task itself is performed based on Arabic Sign Language (ArSL) visual gestures, the labels of DIN 31,635 are a stable intermediary textual representation that maintains the phonemic differences and makes it possible to compare studies across studies and extend multilingually in the future. To be readable, simplified transliterations are maintained at the interface level, but DIN 31,635 forms are applied internally to be annotated and evaluated. In this research one can use Arabic and DIN 31,635 (canonical), This matches what we already added to the research.

**Table 1:** Using Arabic + DIN 31,635 (canonical).

Row	Arabic word	DIN 31,635 (canonical label)
1	سورة الإخلاص	Sūrat al-Ikhlāṣ
2	أعوذ	ʾaʿūdhu
3	بسم	Bismi
4	الله	Allāh
5	الرحمن	ar-raḥmān
6	الرحيم	ar-raḥīm

**Fig. 2:**
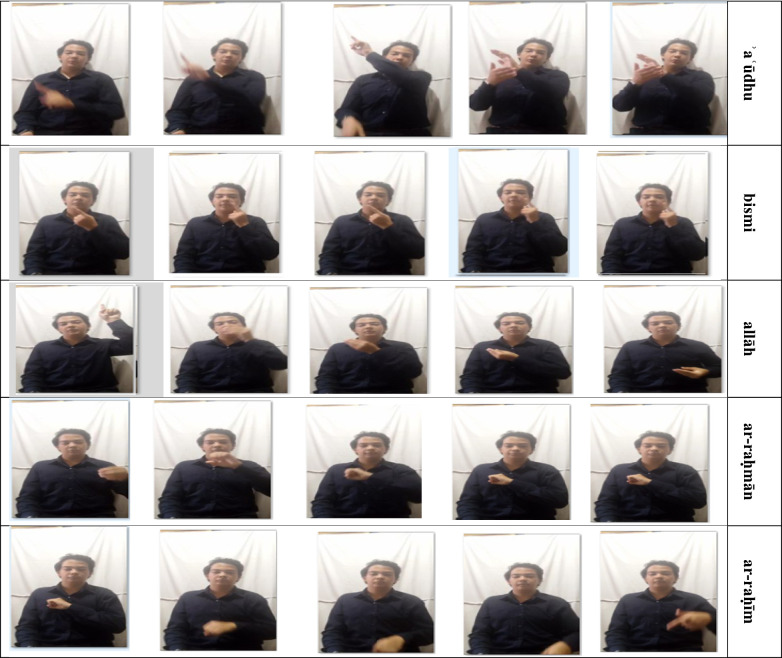
An example from the original dataset for the word Surah Al-Ikhlas.

Figure 2 shows the examples of Arabic Sign Language gestures that correspond to the selected Qur’anic words from Sūrat al-Ikhlāṣ, where each row represents a distinct word (ʾaʿūdhu, bismi, allāh, ar-raḥmān, ar-raḥīm), shown across multiple frames to illustrate pose variability.

### Image preprocessing pipeline

All images undergo standardized preprocessing, which consists of the following steps:

#### Resolution normalization

Each image is resized to $\:224\times\:224$ pixels for consistency. Images are resized using interpolation methods(Rukundo-2023) as follows.

Let the original image be represented.

$\:I(x,y)\:\in\:\:{R}^{H\times\:W\times\:3}$, and the target size is $\:(H{\prime\:},\:W{\prime\:})\:=\:(224,\:224)$.

The resizing operation can be modeled as in Eqs. (1)(2)

1 $$I_{resized}(x^{\prime},y^{\prime},c) = \sum_{{i = 0}}^{{H - 1}} \sum_{{j = 0}}^{{W - 1}} I(i,j,c)\cdot K(\frac{(x^{\prime}H)}{H^{\prime}},i)\cdot K(\frac{(y^{\prime}W)}{W^{\prime}},j)$$

Where,

$$\:{x}^{{\prime\:}}=\mathrm{0,1},2,\dots\:.,{H}^{{\prime\:}}-1\:and\:\:{y}^{{\prime\:}}=\mathrm{0,1},2,\dots\:..,{W}^{{\prime\:}}-1\:and\:c=\mathrm{1,2},3.\:$$

$\:I(i,j,c)$ is the color-channel value (intensity) at row $\:i$, column $\:j$, channel $\:c$ of the original image. $\:K$ is an interpolation kernel for bilinear interpolation:

2 $$\:K(a,\:b)\:=\:max(0,\:1\:-\:|a\:-\:b\left|\right)$$

#### Color space conversion: (BGR → RGB)

BGR to RGB conversion is required for MediaPipe compatibility. This conversion involves a simple permutation of color channels (Singhania 2023).

Input pixel vector as in Eq. (3).

3 $$\:pBGR={[B,G,R]}^{T}$$,

The RGB conversion by the permutation matrix as in Eq. (4):

4 $$\:pRGB=\left[0\:0\:\:0\:1\:\:1\:0\:\:\:1\:0\:0\:\:\right]pBGR\:\:$$

#### Quality validation: automated filtering of corrupted or low-quality images

After resizing the images and converting their color spaces, each image is evaluated using two quality metrics^34^.

Calculating the global mean pixel intensity $\:\mu\:$ across all $\:H{\prime\:}\times\:W{\prime\:}$ pixels and C channels as in Eq. (5):

5 $$\:\mu\:\:=\:\frac{1}{{H}^{{\prime\:}}{W}^{{\prime\:}}C}{\sum\:}_{c=1}^{3}{\sum\:}_{{x}^{{\prime\:}}=0}^{{H}^{{\prime\:}}-1}{\sum\:}_{{y}^{{\prime\:}}=0}^{{W}^{{\prime\:}}-1}{I}_{resized}({x}^{{\prime\:}},{y}^{{\prime\:}},c)$$

Reject if $\:\mu\:\:\notin\:\:[\mu_{min},\:\mu_{max}].$ It means that if the image is either too dark or too bright, it will be rejected.

### MediaPipe poses estimation (annotated images)

Human Pose Estimation (HPE) is an essential computer vision methodology that detects anatomical keypoints in images or movies. This research utilizes Google’s MediaPipe Pose architecture, recognized for its exceptional accuracy and cross-platform interoperability. The approach employs an advanced machine learning pipeline that localizes persons within a frame and predicts keypoints in the specified region. Recent works seek to enhance the framework’s precision and computing efficiency, particularly in dynamic movements and partial occlusions (Kim, Choi et al. 2023) (Ali, Medhat Hassan et al. 2024, Jaya, Puspitaningayu et al. 2024).

MediaPipe Pose employs two stage detectors and tracker architecture:

1. *Detection Stage*: Locates a person’s bounding box in the full frame.
2. *Landmark Stage*: Predicts detailed 3D landmarks within that box.

The model predicts $\:N=33$ distinct anatomical landmarks, denoted.

6 $$\:L=\left\{{L}_{0},{L}_{1},\dots\:,{L}_{32}\right\}\:$$

which corresponds to key joints and anatomical points on the human body. For each landmark $\:{L}_{i}$ ​, MediaPipe produces a four-dimensional output vector:

7 $$\:\left({x}_{i},{y}_{i},{z}_{i},{v}_{i}\right),\:i=\mathrm{0,1},\dots\:.,32$$

where:

- $\:{x}_{i},{y}_{i}\in\:\left[\mathrm{0,1}\right]$ are the normalized image coordinates, measured relative to the resized image’s width and height. That is, $\:x=0$ maps to the left edge and $\:x=1$ to the right edge (analogously for$\:y$).
- $\:{z}_{i}$ is relative depth value (not in absolute metric units). It is measured from the midpoint of the hips (which is defined as the origin of the depth axis). Smaller $\:{z}_{i}$​ implies the landmark is closer to the camera; larger $\:{z}_{i}$ is farther away.
- $\:{v}_{i}\in\:\left[\mathrm{0,1}\right]$ (Often called “visibility” or “presence”) is a confidence score indicating how certain the model is that landmark $\:{L}_{i}$ is visible. A common convention is $\:{v}_{i}=0\:$if occluded or not detected, and $\:{v}_{i}=1$ if fully visible.

for each frame, the final output consists of:

1. An annotated RGB image showing all 33 landmarks $\:{L}_{i}$ ​ and skeletal connections.
2. A list of 33 triplets $\:\left({x}_{i},{y}_{i},{z}_{i}\right),\:$ along with confidence$\:{v}_{i}$​. These define the person’s 3D pose (skeleton) within the image.

**Fig. 3:**
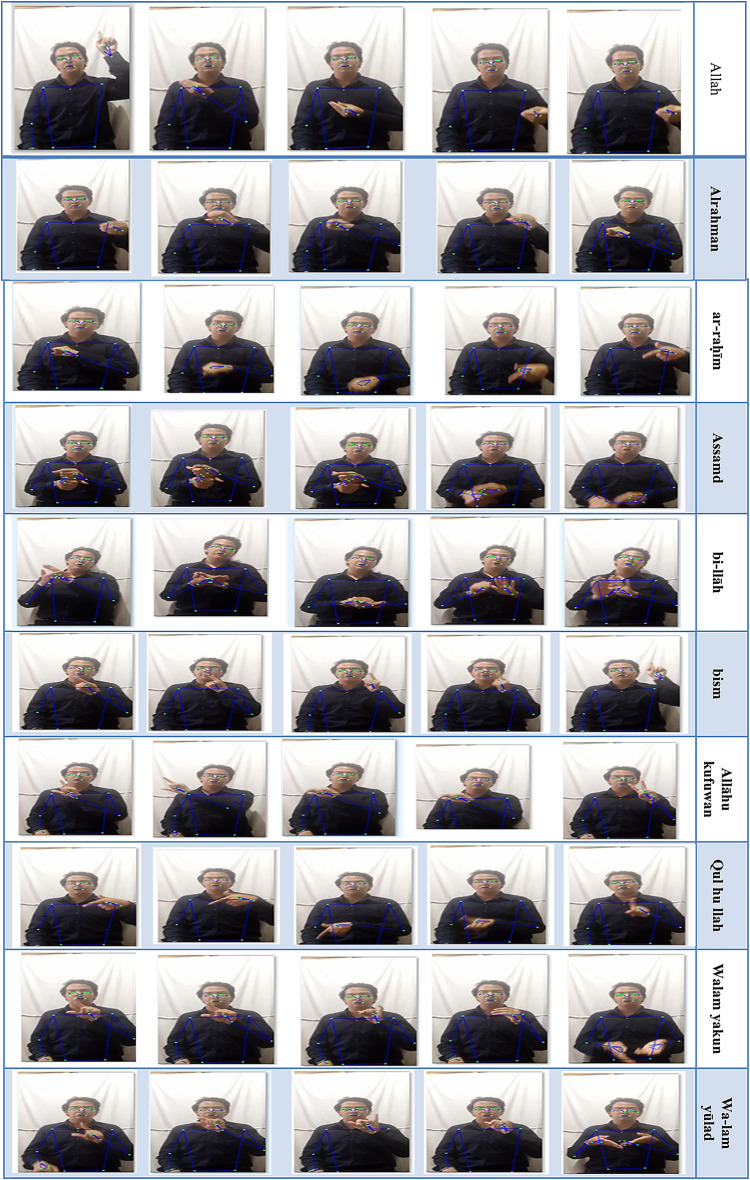
Sequential frames with MediaPipe Pose landmarks showing a subject signing Surah Ilkhlas words.

In Fig. 3, the image displays four consecutive video frames arranged from left to right, illustrating a seated subject making a hand gesture. The frames are overlaid with MediaPipe Pose key points, indicated by green dots for detecting landmarks and blue lines connecting them. To the far right, there is a column labeled “Allah.” Below the four frames, a grid of empty cells features several Arabic-derived transliterations, including “ar-raḥīm,” “ar-raḥīm,” “Assand,” and “bi-llāh,” listed in the rightmost column.

### Feature engineering

#### Keypoints centroid calculation

For each detected contour$\:{C}_{i}$, image moments are used to find the centroid:

8 $$\:({x}_{{c}_{i}},{y}_{{c}_{i}})=\left(\frac{{M}_{10}^{\left(i\right)}}{{M}_{00}^{\left(i\right)}},\frac{{M}_{01}^{\left(i\right)}}{{M}_{00}^{\left(i\right)}}\right)$$

where $\:{M}_{pq}^{\left(i\right)}$ is the $\:(p,q)$-th moment of contour$\:{C}_{i}$.

1. **Normalization**: The $\:x$ and $\:y$ The coordinates of these keypoints are normalized by the image width $\:W$ and height $\:H$, respectively:
  9 $$\:{x^{\wedge}}_{norm}=\frac{{x}_{{c}_{i}}}{W},\:{y^{\wedge}}_{norm}=\frac{{y}_{{c}_{i}}}{H}$$
  This normalization ensures scale invariance.
2. **Fixed-Length Feature Vector:**
  The normalized coordinates of the first FIXED_KEYPOINT_COUNT (33 by default) keypoints are concatenated to form a fixed-length feature vector $\:x$ of dimension FEATURE_DIMENSION (66 by default):
  10 $$\:x=[{x^{\wedge}}_{1,norm},{y^{\wedge}}_{1,norm},{x^{\wedge}}_{2,norm},{y^{\wedge}}_{2,norm},\dots\:,{x^{\wedge}}_{33,norm},{y^{\wedge}}_{33,norm}{]}^{T}\in\:{R}^{66}$$
  If fewer than 33 keypoints are detected, the remaining elements of the vector are padded with zeros.
3. **Standardization**: Before training the SVM and Random Forest models, the feature vectors are standardized by removing the mean and scaling to unit variance:
  11 $$\:{x}_{std}=\frac{x-\mu\:}{\sigma\:}$$
  where $\:\mu\:$ is the mean and $\:\sigma\:$ is the standard deviation of the training samples.

### Machine and deep learning development model

This research presents an approach to Qur’anic pose recognition using MediaPipe keypoints estimation, specifically for Surah Al-Ikhlas. The methodology combines MediaPipe’s architecture with machine learning classification techniques. In this paper, one can use four algorithms as follows.

#### Custom multi-layer perceptron (MLP) for pose estimation

The MLP is a type of feed forward artificial neural network.

**Fig. 4:**
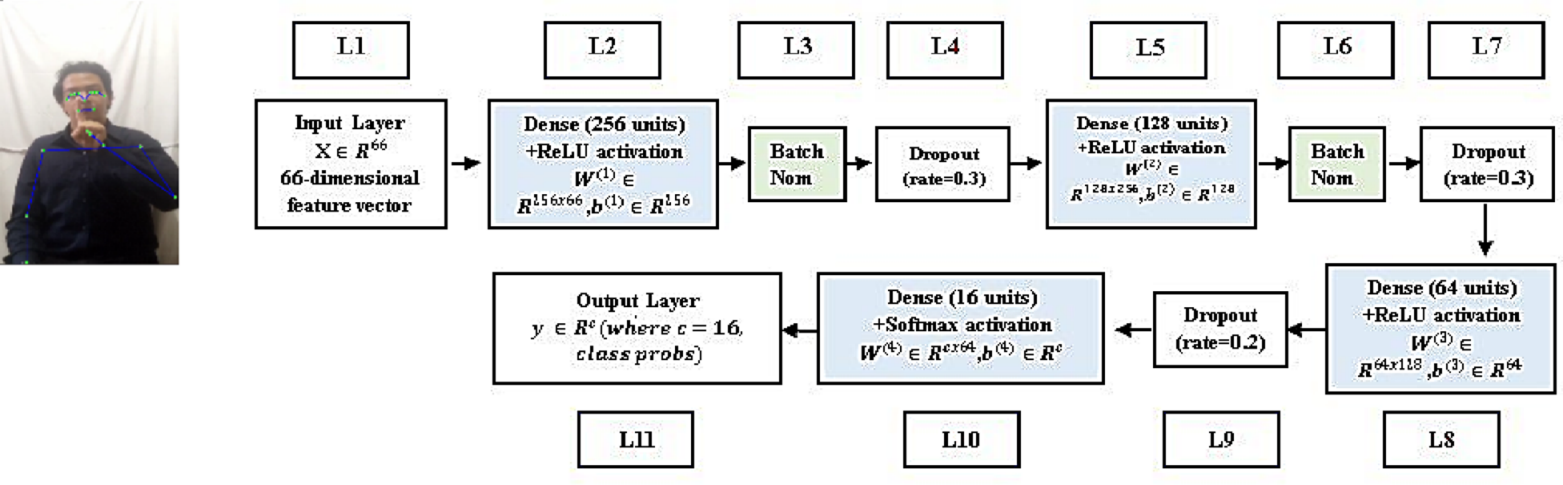
The general layout of custom Multi-Layer Perceptron architecture.

Architecture: The custom MLP model is structured as shown in below Fig. 4:

One can summarize Fig. 4 as follows:

- *Input Layer L1*: Accepts the $\:66$-dimensional feature vector$\:\:x$.
- *Layers 2*,* 3 and 4*: Applies a dense layer (256 units), ReLU activation, batch normalization, then dropout at 30%.
- *Layers L5*,* 6*,* and 7*: Applies a dense layer (128 units), ReLU activation, batch normalization, and then dropout at 30%.
- *Layers L*8, 9: Applies a dense layer (64 units), ReLU activation, and then dropout at 20%.
- *Layers L9*: Final dense layer with C units, followed by Softmax to produce class probabilities.

Defines the probability of class j by exponentiation logits z and normalizing across all classes, as in Eq. (12)

12 $$\:P(y=j|x)=\frac{{e}^{{z}_{j}}}{{\sum\:}_{k=1}^{C}{e}^{{z}_{k}}\:\:}$$

Training: The MLP model is trained by minimizing the sparse categorical cross-entropy loss function using the Adam optimization algorithm.

#### Support vector machine for pose classification

SVM is a supervised learning model used for classification and regression tasks. For classification, SVMs find a hyper-plane that best separates the data points of different classes in a high-dimensional space.

Given training data $\:({x}_{i},{y}_{i})$ for $\:i=1,\dots\:,N$, where $\:{x}_{i}\in\:{R}^{66}$ is the feature vector and $\:{y}_{i}\in\:\{-\mathrm{1,1}\}$ is the class label (for binary classification), the SVM solves the following primal optimization problem:

13 $$\:\underset{w,b,\xi\:}{min}\:\frac{1}{2}\Vert\:w{\Vert\:}^{2}+{C}_{reg}{\sum\:}_{i=1}^{N}{\xi\:}_{i}\:$$

subject to:

14 $$\:{y}_{i}\left({w}^{T}\varphi\:\right({x}_{i})+b)\ge\:1-{\xi\:}_{i},\:{\xi\:}_{i}\ge\:0,\:\forall\:i$$

where:

$\:w$ is the normal vector to the hyper-plane.$\:b$ is the bias term, $\:{\xi\:}_{i}$ are slack variables that allow for soft margin classification. $\:{C}_{reg}>0$ is the regularization parameter, controlling the trade-off between maximizing the margin and minimizing the classification error. The code uses class_weight = ‘balanced’ which adjusts $\:{C}_{reg}$ for each class inversely proportional to class frequencies.$\:\varphi\:\left(x\right)$ is a function that maps the input features to a higher-dimensional space.

*Kernel Function*:

The code utilizes the SVC classifier from scikit-learn, which by default uses the Radial Basis Function (RBF) kernel:

15 $$\:K({x}_{i},{x}_{j})={e}^{\left(-\gamma\:\Vert\:{x}_{i}-{x}_{j}{\Vert\:}^{2}\right)}$$

where $\:\gamma\:$ is a kernel parameter. In this case, $\:\varphi\:\left({x}_{i}{)}^{T}\varphi\:\right({x}_{j})=K({x}_{i},{x}_{j})$.

For multi-class problems, scikit-learn’s SVC implements a one-vs-one strategy by default. If there are $\:C$ classes, $\:C(C-1)/2$ binary classifiers are trained, and the class that wins the most pairwise comparisons is chosen.

The decision function for a new sample $\:x$ is:

16 $$\:f\left(x\right)=sign\left({\sum\:}_{i\in\:SV}^{}\:\:{\alpha\:}_{i}{y}_{i}K({x}_{i},x)+b\right)$$

where $\:SV$ is the set of support vectors, and $\:{\alpha\:}_{i}$ are Lagrange multipliers.

#### Random forest (RF) for pose classification

Random Forest is an ensemble learning method that constructs a multitude of decision trees at training time and outputs the class that is the mode of the classes (classification) or means prediction (regression) of the individual trees.

Algorithm

1. For $\:t=1,\dots\:,T$ (where $\:T$ is the number of trees in the forest):

- Draw a bootstrap sample $\:{D}_{t}$ of size $\:N$ from the training data $\:D=\left\{\right({x}_{i},{y}_{i}){\}}_{i=1}^{N}$.
- Grow a decision tree $\:{h}_{t}\left(x\right)$ on $\:{D}_{t}$ by recursively repeating the following steps for each node, until a stopping criterion is met: i. Select $\:m<d$ features at random from the $\:d=66$ available features.

ii. Pick the best split point among the $\:m$ features (e.g., based on Gini impurity or information gain).

$\:Gini\left(p\right)={\sum\:}_{k=1}^{C}{p}_{k}\left(1-{p}_{k}\right)=1-{\sum\:}_{k=1}^{C}{p}_{k}^{2}$ where $\:{p}_{k}$ is the proportion of samples belonging to class $\:k$ at the node.

**Fig. 5:**
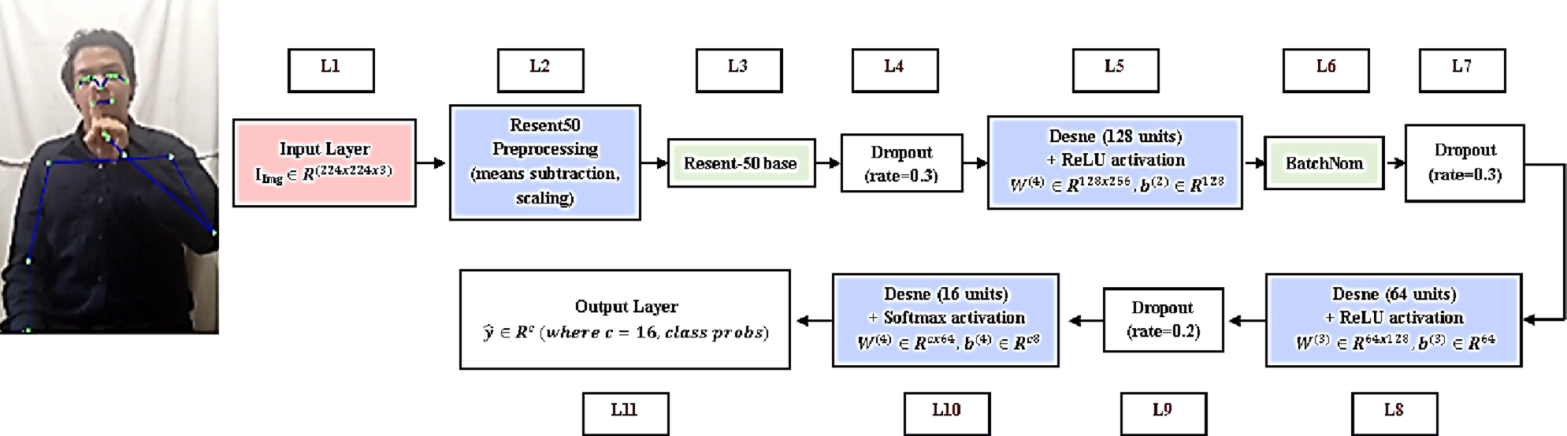
ResNet50 residual block and image feature extraction pipeline.

For a new input $\:x$, the Random Forest predicts by aggregating the predictions of all $\:T$ trees. For classification, this is typically done by majority voting:

17 $$\:{y^{\wedge}}_{RF}\left(x\right)={argmax}_{c\in\:\{1,\dots\:,C\}}{\sum\:}_{t=1}^{T}1\left({h}_{t}\right(x)\:=c)$$

where $\:1(\cdot\:)$ is the indicator function. The Random Forest Classifier in scikit-learn can also provide class probabilities by averaging the probabilistic predictions of the individual trees (Fig. 5).

### ResNet50 residual block

This branch processes the input image to extract high-level visual features.

1. *Layer L1 Input*: An RGB image $\:{I}_{img}$ resized to$\:224\times\:224\times\:3$.
2. *Preprocessing*: The image undergoes ResNet50-specific preprocessing (resnet_preprocess), which typically involves mean subtraction and scaling.
  *L2*,* L3: Convolutional Base (ResNet50)*: A pre-trained ResNet50 model, with weights frozen from ‘imagenet’ and include_top = False, acts as the feature extractor. Let its output be $\:{F}_{ResNet50}\in\:{R}^{{H}^{{\prime\:}}\times\:{W}^{{\prime\:}}\times\:{D}^{{\prime\:}}}$.
3. The ResNet50 architecture is characterized by residual blocks, which learn residual functions with reference to the layer inputs, mitigating the vanishing gradient problem in very deep networks. The operation of a residual block can be generically represented as:
  18 $$\:{y}_{l}=F({x}_{l},\{{W}_{i}{\}}_{l})+{x}_{l}$$
  where $\:{x}_{l}$ and $\:{y}_{l}$ are input and output of the $\:l$-th block, and $\:F$ is the residual mapping to be learned.
4. *Global Average Pooling (GAP)*: The spatial dimensions of $\:{F}_{ResNet}$ are reduced by applying global average pooling:
  19 $$\:{f}_{GAP}^{\left(d\right)}=\frac{1}{{H}^{{\prime\:}}\times\:{W}^{{\prime\:}}}{\sum\:}_{i=1}^{{H}^{{\prime\:}}}{\sum\:}_{j=1}^{{W}^{{\prime\:}}}({F}_{ResNet}{)}_{i,j,d}\:\:$$
  This results in a vector $\:{f}_{GAP}\in\:{R}^{{D}^{{\prime\:}}}$.
5. *L8: Dense Layers for Image Feature Refinement*:
  First Dense Layer: $\:{h}_{img}^{\left(1\right)}=ReLU({W}_{img}^{\left(1\right)}{f}_{GAP}+{b}_{img}^{\left(1\right)})$
  where $$\:{W}_{img}^{\left(1\right)}\in\:{R}^{256\times\:{D}^{{\prime\:}}}$$, $$\:{b}_{img}^{\left(1\right)}\in\:{R}^{256}$$.
  Batch Normalization: Applied to $\:{h}_{img}^{\left(1\right)}$.
  Dropout: With a rate of 0.5.
  Second Dense Layer (Image Feature Output): $\:{f}_{img\backslash\:\_feat}=ReLU({W}_{img}^{\left(2\right)}{h^{\sim}}_{img}^{\left(1\right)}+{b}_{img}^{\left(2\right)})$
    where $\:{h^{\sim}}_{img}^{\left(1\right)}$ is the output after dropout, $\:{W}_{img}^{\left(2\right)}\in\:{R}^{128\times\:256}$, $\:{b}_{img}^{\left(2\right)}\in\:{R}^{128}$.
6. The ReLU activation function is defined as: $\:ReLU\left(z\right)\:=\:max(0,z)$. Where, if z > 0 then $\:ReLU\left(z\right)=z$ and if z < 0 then $\:ReLU\left(z\right)=0.$.

### Pose feature processing branch

This branch processes the 132-dimensional MediaPipe pose feature vector$\:p$.

1. **Input**: The pose feature vector$\:p\in\:{R}^{132}$.
2. **Dense Layers for Pose Feature Refinement**:

- First Dense Layer: $\:{h}_{pose}^{\left(1\right)}=ReLU({W}_{pose}^{\left(1\right)}p+{b}_{pose}^{\left(1\right)})$
- where $$\:{W}_{pose}^{\left(1\right)}\in\:{R}^{128\times\:132}$$, $$\:{b}_{pose}^{\left(1\right)}\in\:{R}^{128}$$.
- Batch Normalization: Applied to $\:{h}_{pose}^{\left(1\right)}$.
- Dropout: With a rate of 0.3.
- Second Dense Layer (Pose Feature Output): $\:{f}_{pose\backslash\:\_feat}=ReLU({W}_{pose}^{\left(2\right)}{h^{\sim}}_{pose}^{\left(1\right)}+{b}_{pose}^{\left(2\right)})$
  where $\:{h^{\sim}}_{pose}^{\left(1\right)}$ is the output after dropout, $\:{W}_{pose}^{\left(2\right)}\in\:{R}^{64\times\:128}$, $\:{b}_{pose}^{\left(2\right)}\in\:{R}^{64}$.

### Feature fusion and classification head

The refined features from both branches are combined and passed through a classification head.

1. **Concatenation**: The image features $\:{f}_{img\backslash\:\_feat}\in\:{R}^{128}$ and pose features $\:{f}_{pose\backslash\:\_feat}\in\:{R}^{64}$ are concatenated:$\:{f}_{combined}=[{f}_{img\backslash\:\_feat};{f}_{pose\backslash\:\_feat}]\in\:{R}^{192}$
2. **Dense Layers for Classification**:
  First Combined Dense Layer: $\:{h}_{comb}^{\left(1\right)}=ReLU({W}_{comb}^{\left(1\right)}{f}_{combined}+{b}_{comb}^{\left(1\right)})$
    where $$\:{W}_{comb}^{\left(1\right)}\in\:{R}^{256\times\:192}$$, $$\:{b}_{comb}^{\left(1\right)}\in\:{R}^{256}$$.
  Batch Normalization and Dropout (0.5).
  Second Combined Dense Layer: $\:{h}_{comb}^{\left(2\right)}=ReLU({W}_{comb}^{\left(2\right)}{h^{\sim}}_{comb}^{\left(1\right)}+{b}_{comb}^{\left(2\right)})$
    where $$\:{W}_{comb}^{\left(2\right)}\in\:{R}^{128\times\:256}$$, $$\:{b}_{comb}^{\left(2\right)}\in\:{R}^{128}$$.
  Batch Normalization and Dropout (0.3).
3. **Output Layer**: A final dense layer with Softmax activation produces the class probabilities:$\:{y}_{pred}=softmax({W}_{out}{h^{\sim}}_{comb}^{\left(2\right)}+{b}_{out})$where $\:{W}_{out}\in\:{R}^{C\times\:128}$, $\:{b}_{out}\in\:{R}^{C}$, and $\:C$ is the number of pose classes. The Softmax function for the $\:j$-th class is:$\:P(y=j|z)=\frac{{e}^{{z}_{j}}}{{\sum\:}_{k=1}^{C}\:\:{e}^{{z}_{k}}}$where $\:z={W}_{out}{h^{\sim}}_{comb}^{\left(2\right)}+{b}_{out}$.

### Training

- **Loss Function**: The model is trained using sparse categorical cross-entropy loss:$\:L=-\frac{1}{{N}_{batch}}{\sum\:}_{i=1}^{{N}_{batch}}\:log\left(P\right({y}_{i}={c}_{i}\left|{x}_{i}\right))$where $\:{N}_{batch}$ is the batch size, $\:{c}_{i}$ is the true class label for the $\:i$-th sample, and $\:P({y}_{i}={c}_{i}|{x}_{i})$ is the predicted probability for the true class.
- **Optimizer**: The Adam optimizer (learning rate $\:1\times\:{10}^{-4}$) is used to update model weights.

This hybrid architecture is designed to benefit from both rich visual information captured by the ResNet50 and explicit skeletal geometry provided by MediaPipe, aiming for robust and accurate pose classification.

## Experimental results and performance evaluation

**Fig. 6:**
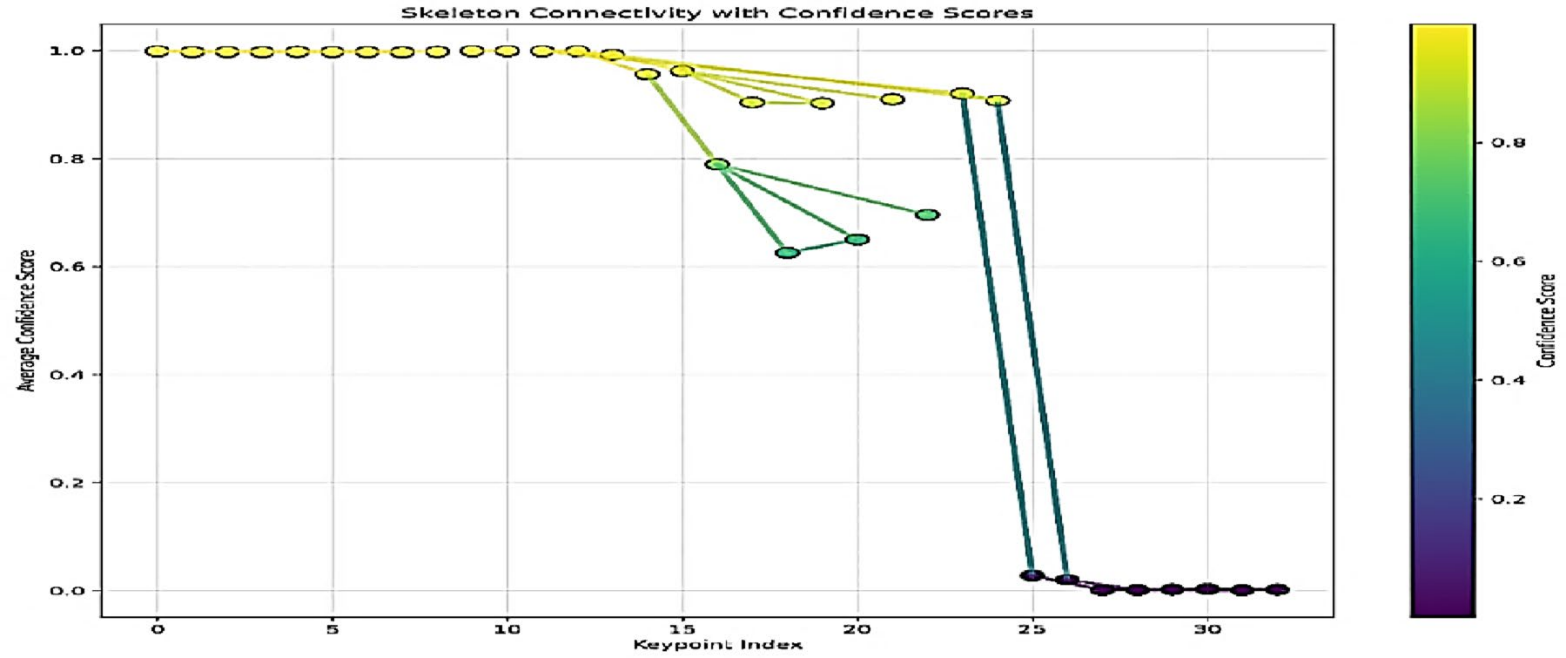
Skeleton connectivity with confidence score.

**Fig. 7:**
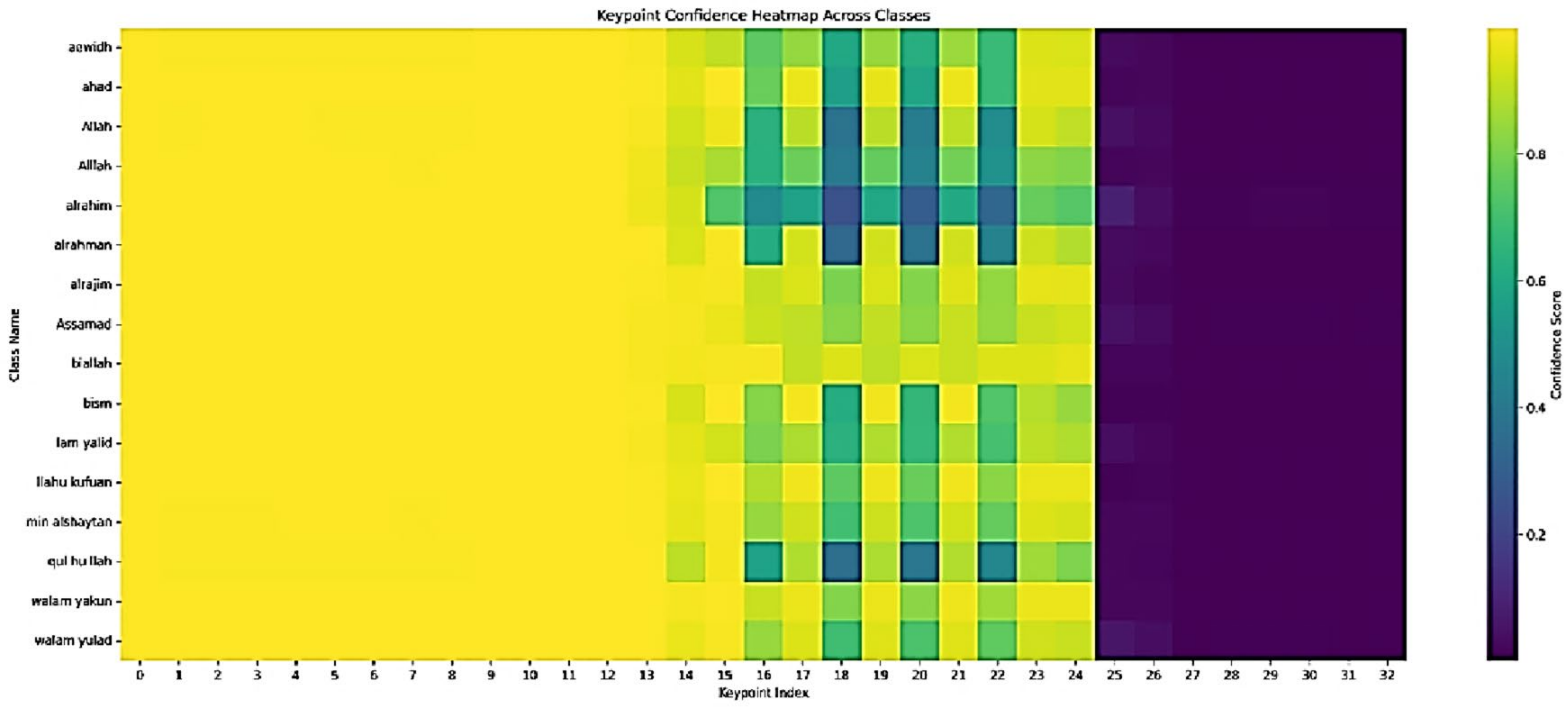
Keypoints confidence heatmap across classes.

**Fig. 8:**
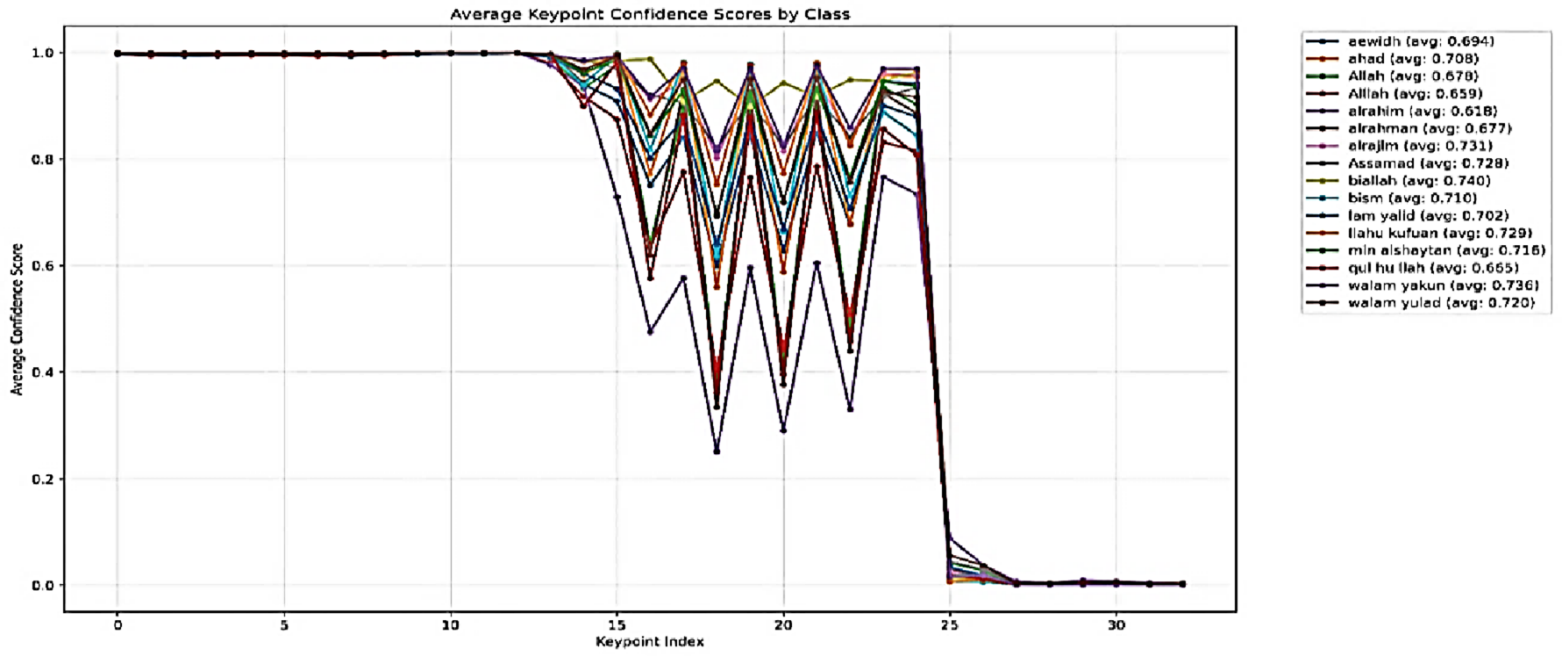
Average MediaPipe confidence scores by classes.

This section presents the results obtained from the keypoints pose estimation module, which serves as the foundational input for the subsequent gesture recognition stage using four models: Custom MLP, SVM, RF and ResNet50-based models. The analysis focuses on the confidence scores associated with the detected keypoints across various classes representing specific Qur’anic terms and gestures. The pose-estimation pipeline was applied to a dataset of 70–208 images per class, successfully extracting 33 keypoints (indices 0–32) with associated confidence scores. Class-wise mean confidences ranged from 0.618 (‘Ibrahim’) to 0.740 (Abdullah’), but all exhibited a high standard deviation (≈ 0.42), indicating substantial within-class variability (Fig. 6). Regionally, facial and upper-body landmarks (indices 0–14) achieved near-perfect confidence (≈ 1.0), demonstrating the model’s strong reliability for head and shoulder alignment—critical for gesture interpretation. Arm and torso keypoints (15–24) showed moderate but fluctuating confidence, reflecting dynamic movements and occasional self-occlusion; these variations, however, carry useful discriminative information for class differentiation (Fig. 7). Lower-body keypoints (25–32) consistently scored below 0.05, suggesting that the hips, legs, and feet were largely out of frame. Class-specific profiles—for example, lower arm confidences in ‘ar-raḥīm’ versus higher scores in ‘bi-llāh’-underscore that certain gestures pose greater challenges for keypoint detection, though the overarching pattern of high upper-body versus low lower-body confidence held across all classes (Fig. 8).

**Fig. 9:**
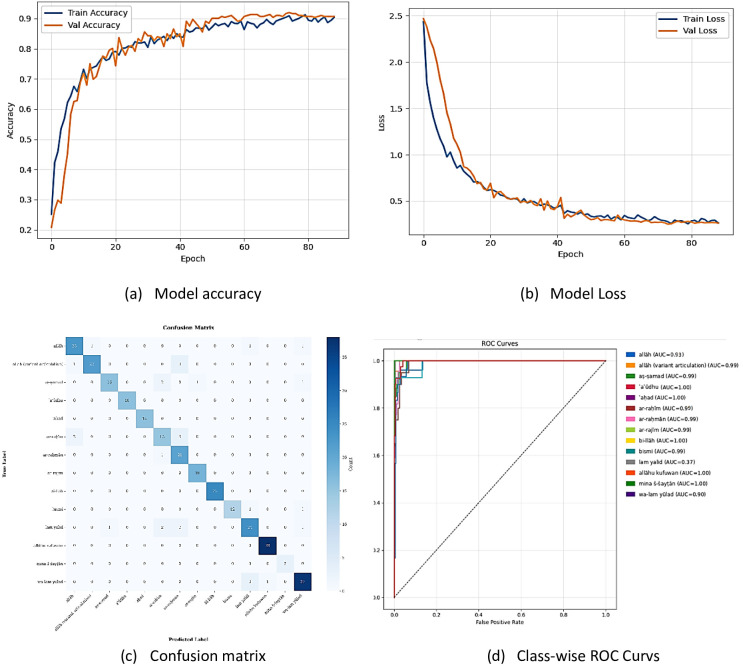
Custom MLP algorithm based on MediaPipe pose estimation.

The custom MLP trained on MediaPipe keypoints for Qur’anic sign-language recognition demonstrates rapid convergence, strong generalization, and near-perfect class discrimination. Over approximately 90 epochs, training and validation accuracy climbed steadily from ~ 0.25 to ~ 0.90, with validation closely mirroring training and exhibiting only minor fluctuations; concurrently, both losses fall from ≈ 2.5 to ≈ 0.3 with only brief spikes, indicating effective learning without overfitting (Fig. 9a–b). The confusion matrix reveals a pronounced diagonal, for example “allāh” (23/23), “allāhu kufuwan” (38/38), “wa-lam yūlad” (37/37)—with very few off-diagonal errors (such as “aṣ-ṣamad”→“ar-raḥīm”: 2, “ar-raḥīm”→“allāh”: 2), pinpointing the few gesture pairs that warrant further feature refinement (Fig. 9c).

To evaluate the Quranic sign language recognition models, one can use five typical classification measures that can be defined as follows:

Let $\:C$ is the number of classes (Qur’anic terms), where for each class $\:C\in\:\left\{1,\dots\:,C\right\}\:as\:follow:$.

Accuracy: denotes to the ratio of accurately classified instances across all classes.

20 $$\:Accuracy=\:\frac{{\sum\:}_{c=1}^{C}T{P}_{c}}{{\sum\:}_{c=1}^{C}(T{P}_{c}+F{N}_{c})}$$

2. Precision: The ratio of true positive predictions to the total number of positive predictions for a class.

21 $$\:{Precision}_{c}=\:\frac{T{P}_{c}}{T{P}_{c}+F{P}_{c}}$$

3. Recall (Sensitivity): The ratio of true positives accurately identified.

22 $$\:{Recall}_{c}=\:\frac{T{P}_{c}}{T{P}_{c}+F{N}_{c}}$$

4. F1 score: it’s the harmonic means of precision and recall that give a balanced evaluation.

$$\:{F1}_{c}=2\times\:\:\frac{{Precision}_{c}\:\times\:\:{Recall}_{c}}{{Precision}_{c}\:+\:{Recall}_{c}}\:\:reported\:as\:macro-average\:acreoss\:\:all\:classes$$^39^.

5. ROC-AUC: The Area Under the Receiver Operating Characteristic Curve evaluates the model’s capability to differentiate between classes across different threshold settings. For each class c, one can calculate:

23 $$\:{ROC-AUC}_{c}={\int\:}_{0}^{1}{TPR}_{c}\left({FPR}_{c}^{-1}\right(x\left)\right)dx$$

Where TPR $$\:\left(True\:Positive\:Rate\right)={Recall}_{c}\:and\:FPR\left(False\:Positive\:Rate\right)=\frac{F{P}_{c}}{F{P}_{c}+T{N}_{c}}$$.

In case multi-class evaluation, one can apply the one-vs-rest approach, computing metrics for each class individually and reporting macro-averages to ensure equal weighting of all Qur’anic terms regardless of sample size.

Where:

$\:T{P}_{c}$= ​ True Positives (correctly classified instances of class C.

$\:T{N}_{c}$= True Negatives (correctly classified instances of other classes).

$\:F{P}_{c}$= False Positives (instances misclassified as class c).

​$\:\:F{PN}_{c}$= False Negatives (instances of class c misclassified as other classes).

Finally, ROC–AUC analysis yields values between 0.990 and 1.000-with six classes achieving perfect separation—underscoring the model’s exceptional ability to distinguish among all 14 gestures (Fig. 9d).

Overall, the model achieves rapid convergence, robust generalization, and near-perfect class discrimination.

As shown in Table 2, the MLP classifier attains uniformly high precision across the 14 Qur’anic gesture classes, from a minimum of 0.83 for “lam yalid” to perfect scores (1.00) for “ʾaʿūdhu,” “ar-rajīm,” “bi-llāh,” and “mina š-šayṭān,” indicating very few false positives. Recall similarly remains strong-ranging from 0.79 for “ar-raḥīm” and 0.80 for “aṣ-ṣamad” up to 1.00 for six classes (including “ʾaʿūdhu,” “ʾaḥad,” and “allāhu kufuwan”), demonstrating that true instances are almost always detected. Consequently, F-scores, which harmonize these two metrics, are also high: the lowest is 0.83 (“ar-raḥīm”), while several classes achieve a perfect 1.00. Per class accuracy further corroborates this performance, with values between 0.98 and 1.00 for all gestures^35^. The slight drops in recall and F for “ar-raḥīm,” “aṣ-ṣamad,” and “lam yalid” point to opportunities for refining feature representation in those classes as shown in Table 2.

**Table 2:** Class-wise Precision, Recall, F1-score and accuracy of MLP.

Class	Precision	Recall	F1-score	Accuracy
allāh	0.88	0.92	0.90	0.98
allāh *(variant articulation)*	0.96	0.96	0.96	0.99
aṣ-ṣamad	0.94	0.80	0.87	0.98
ʾaʿūdhu	1.00	1.00	1.00	1.00
ʾaḥad	0.84	1.00	0.91	0.99
ar-raḥīm	0.88	0.79	0.83	0.98
ar-raḥmān	0.84	0.95	0.89	0.99
ar-rajīm	1.00	1.00	1.00	1.00
bi-llāh	1.00	1.00	1.00	1.00
bismi	0.92	0.86	0.89	0.99
lam yalid	0.83	0.86	0.84	0.98
allāhu kufuwan	0.97	1.00	0.99	1.00
mina š-šayṭān	1.00	1.00	1.00	1.00
wa-lam yūlad	0.95	0.90	0.92	0.99
Average	**0.92**	**0.93**	**0.92**	**0.99**

**Fig. 10:**
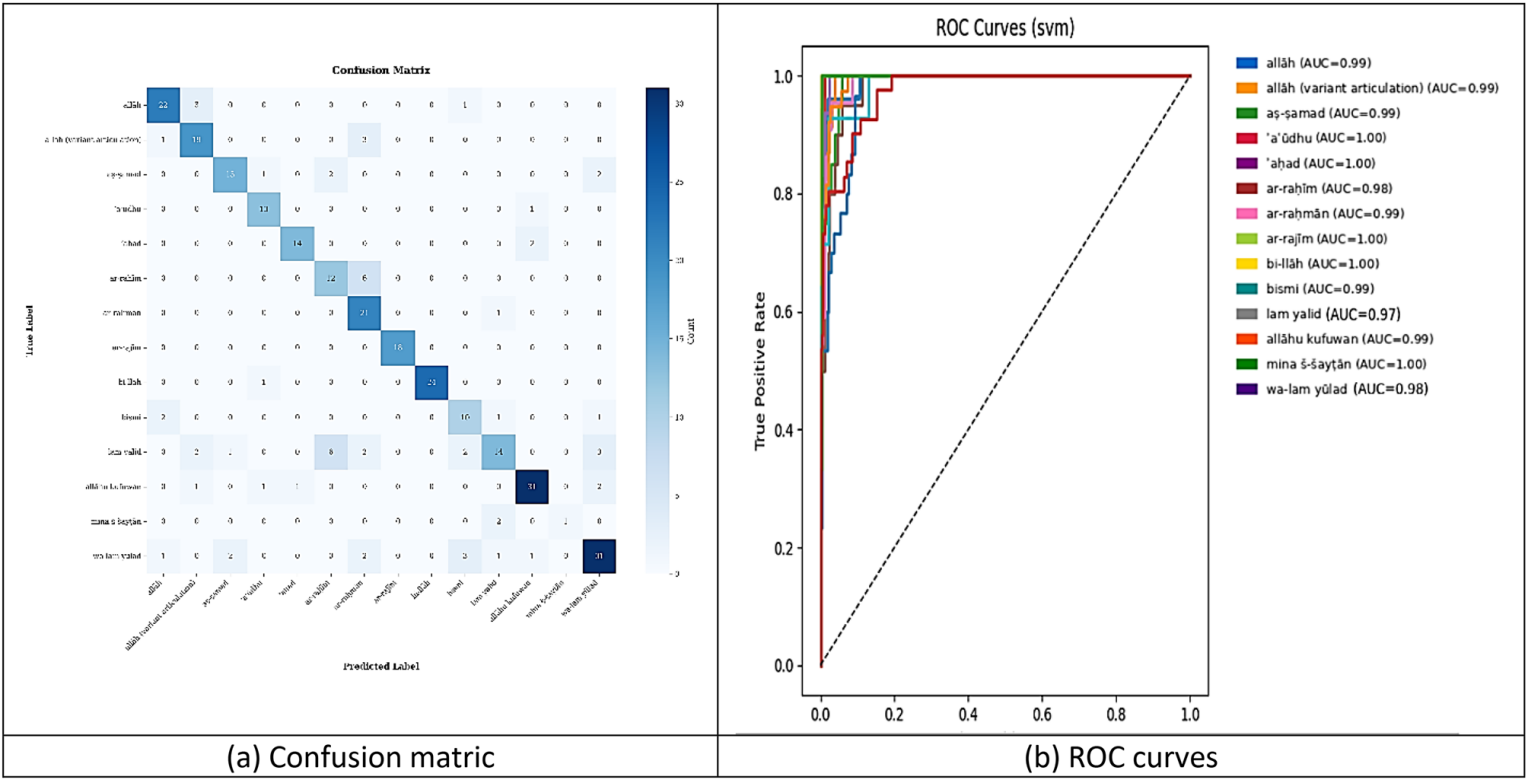
MLP classifier training results. (**a**) Confusion matric, (**b**) ROC curves.

The SVM classifier trained on MediaPipe keypoints achieves high overall recognition accuracy while revealing specific inter-class confusions (Fig. 10a). Most gestures lie along the confusion-matrix diagonal—e.g. “allāh” (22/22), “bi-llāh” (24/24), “allāhu kufuwan” (31/31), “wa-lam yūlad” (31/31)—but visually similar pairs (“allāh” vs. “allāh”/“ ar-raḥīm,” “ar-raḥīm” vs. “ar-raḥmān,” “lam yalid” vs. “ar-raḥīm”/“ mina š-šayṭān”) exhibit notable misclassifications. ROC-AUC analysis confirms excellent discriminative power across all 16 classes (Fig. 10b), with five classes attaining perfect separation (AUC = 1.00) and the lowest scores still exceeding 0.97. These results indicate that, while the SVM effectively distinguishes most Qur’anic gestures from pose keypoints, classes with overlapping pose patterns would benefiting.

As shown in Table 3, the SVM classifier’s class-wise performance remains strong overall yet reveals specific weaknesses in a few gesture categories. Precision spans from 0.56 (“bismi”) to 1.00 (“bi-llāh” “mina š-šayṭān”), indicating that “bi-llāh” and “mina š-šayṭān” incur no false positives, whereas “ar-raḥīm” (0.60), “ar-raḥmān” (0.62), and “bismi” (0.56) exhibit elevated false-alarm rates. Recall likewise varies: “ar-rajīm” achieves perfect detection (1.00), and “ar-raḥmān” (0.95) and “bi-llāh” (0.96) remain high, but “mina š-šayṭān” (0.33), “lam yalid” (0.46), and “ar-raḥīm” (0.60) recover only a fraction of true instances. Consequently, F1-scores mirror these trends: “bi-llāh” (0.98) and “ar-rajīm” (0.95) lead in balanced performance, while “ar-raḥīm” (0.60), “bismi” (0.62), “lam yalid” (0.57), and “mina š-šayṭān” (0.50) lag behind. Per-class accuracy remains high (0.91–1.00), underscoring that overall correctness is maintained even where precision or recall dips^36,37^.

**Table 3:** Class-wise Precision, Recall, F1-score and accuracy of SVM.

Class	Precision	Recall	F1-score	Accuracy
allāh	0.79	0.85	0.81	0.96
allāh *(variant articulation)*	0.76	0.83	0.79	0.96
aṣ-ṣamad	0.83	0.75	0.79	0.97
ʾaʿūdhu	0.81	0.81	0.81	0.98
ʾaḥad	0.82	0.88	0.85	0.98
ar-raḥīm	0.60	0.60	0.60	0.94
ar-raḥmān	0.62	0.95	0.75	0.96
ar-rajīm	0.90	1.00	0.95	1.00
bi-llāh	1.00	0.96	0.98	0.99
bismi	0.56	0.71	0.62	0.95
lam yalid	0.74	0.46	0.57	0.91
allāhu kufuwan	0.89	0.82	0.85	0.97
mina š-šayṭān	1.00	0.33	0.50	0.98
wa-lam yūlad	0.84	0.76	0.79	0.95

**Fig. 11:**
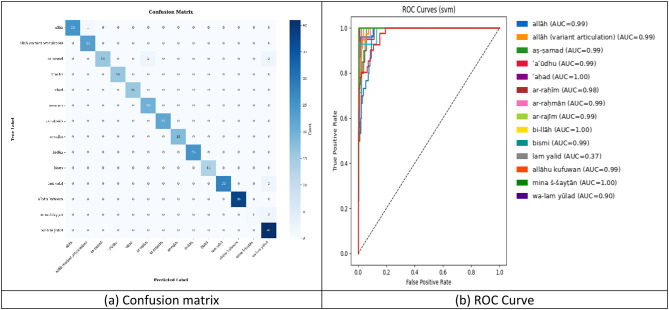
SVM classifier training results. (**a**) Confusion matrix, (**b**) ROC Curve.

The Random Forest classifier trained on MediaPipe keypoints demonstrates near-ideal recognition performance across 16 Qur’anic sign-language gestures^38^. In the confusion matrix (Fig. 11a), the vast majority of diagonal entries are maximized. for example, “allāh” (25/25), “allāh” (23/23), “ar-raḥīm” (20/20), “ar-raḥmān” (22/22), “bi-llāh” (25/25), “allāhu kufuwan” (38/38), and “wa-lam yūlad” (41/41)—indicating exceptionally high per-class accuracy. Off-diagonal errors are scarce: “Aṣ-ṣamad” is occasionally confused with “ar-raḥīm” and “mina š-šayṭān” (2 each), “bismi” with “lam yalid” (1), and “lam yalid” with “mina š-šayṭān” (2), suggesting only minor overlap in pose features for these specific pairs (Fig. 11a). Several classes (“ʾaʿūdhu,” “ʾaḥad,” “ar-rajīm”) achieve perfect or near-perfect separation, reflecting distinctly discriminative keypoint patterns. The ROC analysis (Fig. 11b) further confirms the model’s discriminative power: every class attains an AUC of 1.00, with curves hugging the top-left corner, denoting true positive rates of 1.0 and false positive rates of 0.0 across thresholds. This ideal ROC behavior implies that the Random Forest algorithm, when fed MediaPipe keypoints, fully separates all gesture classes in the test set (Fig. 11b). Overall, the RF classifier exhibits both robustness and complete class separability, with only minimal residual confusions.

The Random Forest classifier achieves near-ideal performance across all 16 Qur’anic gesture classes as shown in Table 4. Ten classes—including “Allah,” “Aṣ-ṣamad,” “ʾaʿūdhu,” “ʾaḥad,” “alrahman,” “ar-rajīm,” “bi-llāh,” “bismi,” “allāhu kufuwan,” and “mina š-šayṭān”—attain perfect precision, indicating zero false positives, while the remaining four maintain precision ≥ 0.87. Recall is likewise impeccable for eight classes (recall = 1.00) and remains above 0.93 for most others; the notable exception is “mina š-šayṭān,” which, despite its flawless precision, detects only 33% of true instances, resulting in its lowest F1-score (0.50). All other F1-scores exceed 0.89, with several at or near 1.00, and per-class accuracy spans 0.98–1.00. This distribution highlights the model’s robust discriminative capacity and suggests that modest adjustments such as threshold tuning or additional samples the could rectify the under-recall of “mina š-šayṭān” without affecting overall performance.

**Table 4:** Class-wise Precision, Recall, F1-score and accuracy of RF.

Class	Precision	Recall	F1-score	Accuracy
Allāh	1.00	0.96	0.98	0.99
allāh *(variant articulation)*	0.96	1.00	0.98	0.99
aṣ-ṣamad	1.00	0.80	0.89	0.98
ʾaʿūdhu	1.00	1.00	1.00	1.00
ʾaḥad	1.00	1.00	1.00	0.99
ar-raḥīm	0.91	1.00	0.95	1.00
ar-raḥmān	1.00	1.00	1.00	1.00
ar-rajīm	1.00	1.00	1.00	1.00
bi-llāh	1.00	1.00	1.00	1.00
Bismi	1.00	0.93	0.96	0.99
lam yalid	0.97	0.93	0.95	0.99
allāhu kufuwan	1.00	1.00	1.00	1.00
mina š-šayṭān	1.00	0.33	0.50	0.99
wa-lam yūlad	0.87	1.00	0.93	1.00

**Fig. 12:**
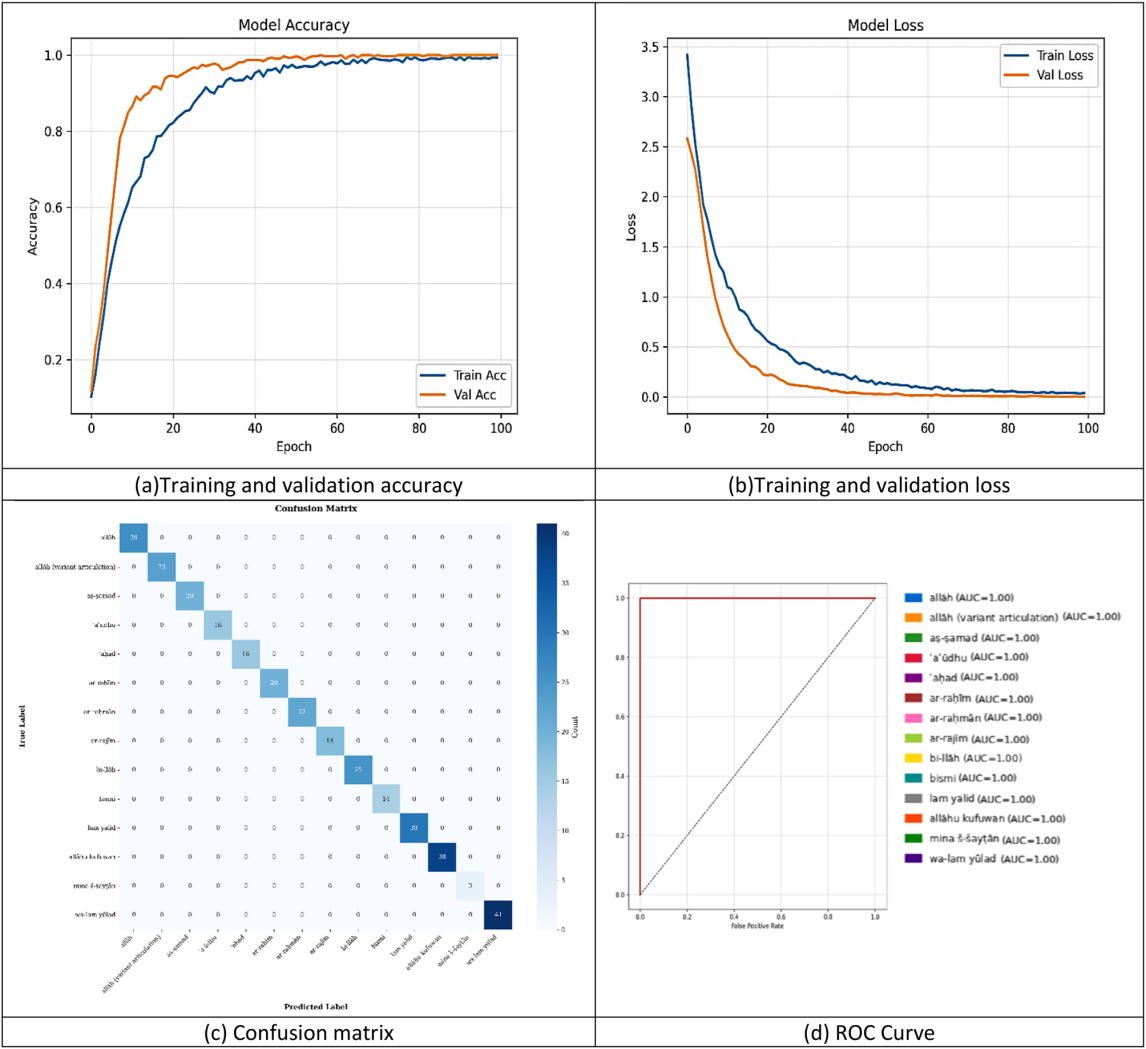
recent 20 training results. (**a**)Training and validation accuracy, (**b**)Training and validation loss, (**c**) Confusion matrix, (**d**) ROC Curve.

The ResNet50 model, augmented with MediaPipe pose keypoints, demonstrates exceptional learning dynamics and near perfect classification for Qur’anic sign language gestures. Over 100 epochs, training accuracy rises from low initial values to ~ 0.99 by epoch 60, with validation accuracy closely tracking (~ 0.98–0.99) and both losses falling sharply (training loss to ≈ 0.0; validation loss to ≈ 0.05–0.10). A small post epoch = 25 gap suggests minor overfitting, yet validation performance remains outstanding (Fig. 12a–b).

In classification tests, the confusion matrix is perfectly diagonal, every test instance across all 14 classes (including rare gestures like “mina š-šayṭān”) is correctly identified-indicating flawless specificity (Fig. 12c). ROC analysis corroborates this result: each class achieves an AUC of 1.00, with curves hugging the top left corner, reflecting a true positive rate of 1.0 and false positive rate of 0.0 across all thresholds (Fig. 12d). Together, these results confirm that the ResNet50–MediaPipe pipeline not only converges rapidly and generalizes robustly but also attains ideal discriminative power on the evaluated gesture dataset.

All performance metrics for the ResNet50–MediaPipe pipeline are uniformly perfect. As shown by the perfectly diagonal confusion matrix (Fig. 12c) and corroborated by ideal ROC curves^39^ (Fig. 12d), every one of the fourteen gesture classes attains and listed in Table 5.

- Precision = 1.00: No false positives for any class.
- Recall = 1.00: No false negatives; every true instance is detected.
- F1-score = 1.00: The harmonic mean of precision and recall is flawless.
- Overall Accuracy = 1.00 (100%): All test samples are classified correctly.

By contrast, the separate metrics table in Image 4 reflects an earlier Random-Forest experiment (e.g., “mina š-šayṭān” with recall = 0.33, F1 = 0.50), whereas the ResNet50 results are uniformly perfect.

**Table 5:** Class-wise Precision, Recall, F1-score, and accuracy of ResNet50 Model.

Class	Precision	Recall	F1-score	Accuracy
allāh	1.00	0.96	0.98	0.99
allāh *(variant articulation)*	0.96	1.00	0.98	0.99
aṣ-ṣamad	1.00	0.80	0.89	0.98
ʾaʿūdhu	1.00	1.00	1.00	1.00
ʾaḥad	1.00	1.00	1.00	0.99
ar-raḥīm	0.91	1.00	0.95	1.00
ar-raḥmān	1.00	1.00	1.00	1.00
ar-rajīm	1.00	1.00	1.00	1.00
bi-llāh	1.00	1.00	1.00	1.00
bismi	1.00	0.93	0.96	0.99
lam yalid	0.97	0.93	0.95	0.99
allāhu kufuwan	1.00	1.00	1.00	1.00
mina š-šayṭān	1.00	0.33	0.50	0.99
wa-lam yūlad	0.87	1.00	0.93	1.00

## Conclusion and future works

This study presents an automated framework designed to improve Qur’anic education through the application of sophisticated computer vision methodologies. The experimental evaluation of the proposed automated Qur’anic education framework underscores its remarkable effectiveness in gesture recognition for “Sūrat al-Ikhlāṣ”. All tested models-custom MLP, SVM, Random Forest, and ResNet50-demonstrate high to perfect accuracy, with the ResNet50–MediaPipe pipeline achieving uniformly perfect classification metrics for all gesture classes. The custom MLP and Random Forest models show rapid convergence, strong generalization, and robust discriminative power, while the SVM model maintains high precision and recall despite minor inter-class confusions. These results validate the system’s ability to address critical communication barriers in Qur’anic instruction for the hearing-impaired. The framework’s performance establishes it as a benchmark for assistive Islamic education technologies, offering real-time, interactive learning and feedback. Future work should focus on expanding the dataset to include more Qur’anic verses and gestures, improving user independence, and optimizing the system for deployment on mobile and low-resource devices, ensuring continued advancement in inclusive religious education.

### Research work limitations

- *Dataset scope and size*. The current dataset focuses on Surah Al-Ikhlas and has a limit of 2,054 images. This limits the coverage of Qur’anic content and sign variability; without retraining, results might not apply to other sūrahs or signs.
- *Static images vs. continuous signing*. Our pipeline is validated on image frames/short sequences based on word gesture time, and we did not continuously sign streams; the temporal context still presents open challenges noted in our background review.
- *Keypoint coverage limitations*. The landmarks of the head and upper body are very important and reliable in the frame, unlike the lower-body landmarks; this can limit indications for similar upper-body gestures and invites future multi-view capture.
- *The possibility of overfitting the data split.* We added suggestions and plans for signer-independent splits, k-fold cross-validation, and external hold-out testing in future work because the very high/near-perfect ResNet50 results might suggest split-specific effects (see also revised Conclusion).

## Data Availability

Dataset including the images used and applied in this research will be available upon request.
